# Fear expression is suppressed by tyrosine administration

**DOI:** 10.1038/s41598-019-52610-x

**Published:** 2019-11-05

**Authors:** Alessandro Soranzo, Luca Aquili

**Affiliations:** Department of Psychology, Sociology and Politics, Sheffield Hallam University, Sheffield, UK

**Keywords:** Fear conditioning, Human behaviour

## Abstract

Animal studies have demonstrated that catecholamines regulate several aspects of fear conditioning. In humans, however, pharmacological manipulations of the catecholaminergic system have been scarce, and their primary focus has been to interfering with catecholaminergic activity *after* fear acquisition or expression had taken place, using L-Dopa, primarily, as catecholaminergic precursor. Here, we sought to determine if putative increases in presynaptic dopamine and norepinephrine by tyrosine administered *before* conditioning could affect fear expression. Electrodermal activity (EDA) of 46 healthy participants (24 placebo, 22 tyrosine) was measured in an instructed fear task. Results showed that tyrosine abolished fear expression compared to placebo. Importantly, tyrosine did not affect EDA responses to the aversive stimulus (UCS) or alter participants’ mood. Therefore, the effect of tyrosine on fear expression cannot be attributed to these factors. Taken together, these findings provide evidence that the catecholaminergic system influences fear expression in humans.

## Introduction

Pavlovian fear conditioning is a valuable behavioural paradigm suitable for neurobiological and physiological analyses^1^ in both animals and humans. Typical fear conditioning protocols in animals consist of presenting a conditioned stimulus (CS) followed by an aversive stimulus (UCS), in a process described as fear *acquisition*. In humans, fear conditioning protocols can also be “instructed” where explicit instructions about CS-UCS contingencies are provided prior to testing. To distinguish between uninstructed and instructed protocols, some authors have suggested to refer to the former as measuring fear *acquisition* and to the latter as fear *expression*^2^, in that learning can already take place in the instruction phase. In the animal literature, however, the term *fear expression* is used to describe conditioned responses (CR) that have developed *following* fear acquisition when the CS is presented in the absence of the UCS.

Using the Pavlovian paradigm, a growing body of literature has indicated the catecholaminergic system as playing a significant role in the acquisition, expression and extinction of fear responses. Animal studies have demonstrated the involvement of dopaminergic D1, D2, D3 and D4 receptors in the amygdala in fear conditioning. D1 and D2 antagonists block the acquisition of fear memories^3–7^ whilst the administration of D1 or D2 agonists increase fear expression^8,9^. Blockade of D3 receptors has, surprisingly, shown to enhance fear acquisition^10^, whilst D4 activation potentiates acquisition^11^. In humans, the effects of a dopamine reuptake blocker (Ritalin) or a dopamine precursor (L-Dopa) have only been investigated with respect to fear extinction and spontaneous recovery^12,13^, but not fear acquisition.

Animal studies have also revealed an important role of norepinephrine (NE) in fear conditioning (for an extensive review, please see^14^). Administration of the non-selective β-adrenergic receptor antagonist propanol prior to training impairs fear acquisition^15,16^. Similarly, administration of the selective α2-adrenergic agonist dexmedetomidine (which decreases activity of NE neurons), weakened cued conditioning^17^. Genetic studies in which NE was reduced, enhanced, or eliminated as in knockout investigations have revealed a more complex picture with respect to the role of NE in fear acquisition^16–18^.

Overall, despite some complex interaction effects, there is robust evidence to suggest that dopamine and norepinephrine are involved in the acquisition/expression of fear responses.

In humans, investigations of dopamine and norepinephrine have been scarce, and have largely focused on the study of extinction, extinction consolidation and reconsolidation processes^19–28^, or in those with posttraumatic stress disorder^29,30^. In these studies, catecholaminergic activity was manipulated *after* fear conditioning had taken place. However, given the existing animal literature, catecholaminergic activity may also be important in regulating fear acquisition and/or expression. To understand whether fear expression is affected, one would need to manipulate dopamine and norepinephrine tone *before* conditioning. Moreover, previous studies were limited to testing the effects of L-Dopa as a catecholaminergic precursor.

In this research, we set out to answer: (i) whether alterations in catecholaminergic tone *before* conditioning would affect fear expression and (ii) whether a different catecholaminergic precursor to L-Dopa namely tyrosine would modulate this effect.

There are good theoretical reasons for the use of tyrosine rather than L-Dopa. The conversion mechanism of tyrosine to dopamine and norepinephrine is restricted to by competition from other endogenous amino acids and by the rate-limiting tyrosine-hydroxylase enzyme whilst L-Dopa is not^31^. As a result, these restrictions would limit the overall enhancement in dopamine and norepinephrine levels from tyrosine. This is important given that low or high doses of drugs that alter dopamine and norepinephrine levels enhance or impair fear conditioning respectively^32,33^.

Here, in a randomized, double-blind study, we tested the effects of tyrosine (placebo controlled) administered *before* fear conditioning. To test the experimental hypothesis that administration of tyrosine would enhance fear expression, skin conductance responses (SCR) were measured in 46 healthy volunteers while undertaking a fear conditioning task.

## Results

### SCR magnitudes in fear expression

Skin conductance responses (SCR) have been used to measure the effects of tyrosine and of placebo (cellulose) administration in a fear conditioning task. Drug administration occurred 60 minutes before fear conditioning. The task had a fear expression phase during which a conditioned stimulus (CS+) predicted the occurrence of an unconditioned stimulus (UCS: 75 db loud beep played through a pair of earphones) whereas another conditioned stimulus predicted the occurrence of a neutral event (CS−, which was a fixation dot, Fig. 3A). CS+ and CS− were paired six times. Responses were analysed on a trial by trial basis.

**Figure 1:**
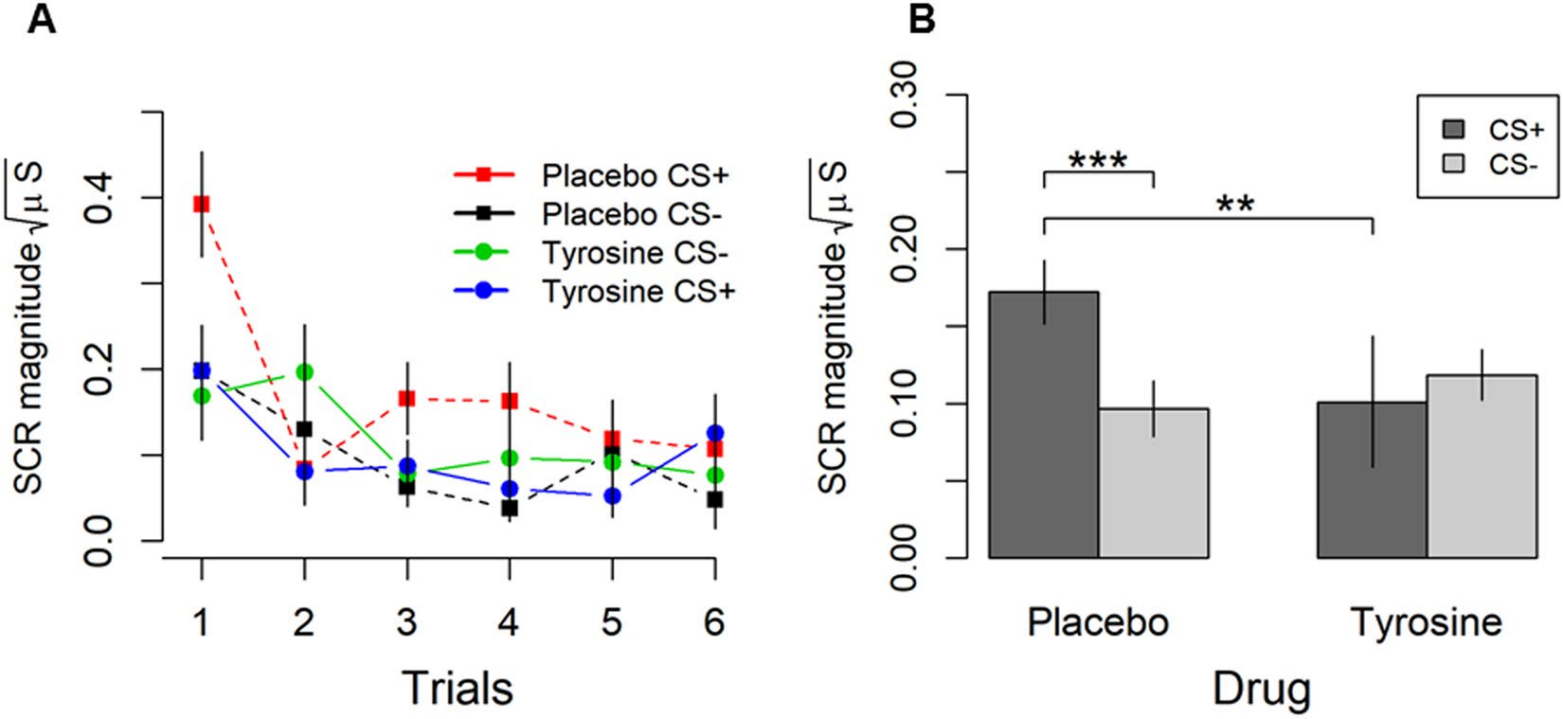
(**A**) Line chart representing skin conductance responses measured in magnitudes across trials (1–6), based on SCR responses to CS+ and CS− in the placebo and tyrosine groups during the fear expression phase. (**B**) Bar chart highlighting the stimuli × drugs significant interaction. Vertical lines represent standard error of the mean. *p = <0.05, **p =  < 0.01, ***p = 0.001.

A 2 × 2 × 6 factorial mixed analysis of variance was conducted on SCR magnitudes during the fear expression phase. The between group variable, *drugs*, having two levels (placebo, tyrosine) the first within subject variable, *stimulus*, having two levels (CS+, CS−), and the second within subject variable, *trial*, having six levels (trial 1–6). There was neither a significant main effect of drugs, *F* (1, 44) = 0.721, *p* = 0.400, η^2^ = 0.016, nor a main effect of stimuli, *F* (1, 44) = 1.678, *p* = 0.202, η^2^ = 0.037, nor a trial × drugs, *F* (5, 44) = 1.781, *p* = 0.118, η^2^ = 0.039, nor a trial × drugs × stimuli significant interaction, *F* (5, 44) = 0.664, *p* = 0.651, η^2^ = 0.015.

There was, however, a significant main effect of trial, *F* (1, 44) = 9.342, *p* < 0.001, η^2^ = 0.175, a significant trial × stimulus interaction, *F* (3.8, 167) = 3.719, *p* = 0.017, η^2^ = 0.067, and most importantly, a significant drugs × stimuli significant interaction, *F* (5, 44) = 4.306, *p* = 0.044, η^2^ = 0.089 (see Fig. 1A).

**Figure 2:**
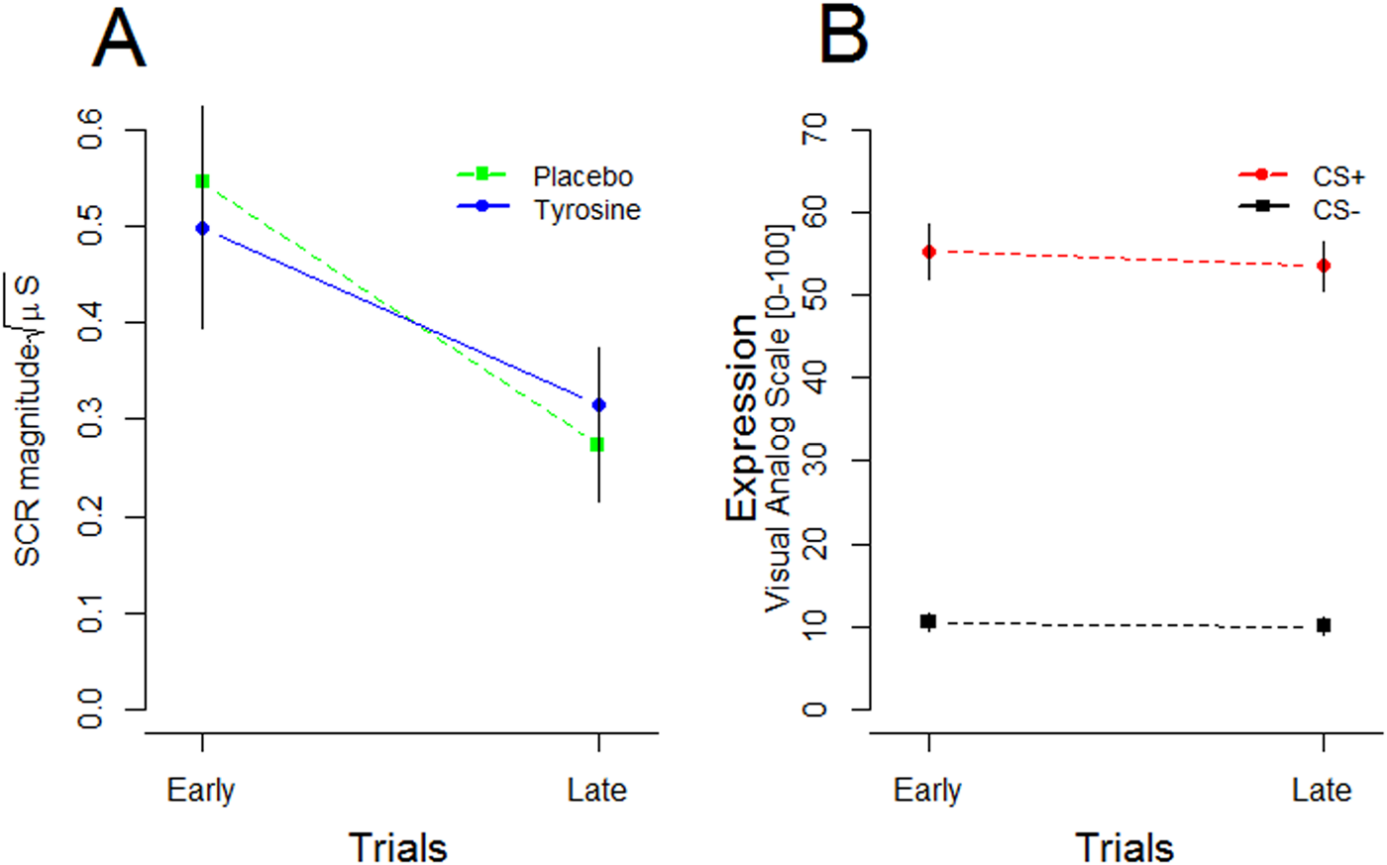
(**A**) Line chart representing skin conductance responses to UCS measured in magnitudes during early and late trials in the placebo and tyrosine groups. (**B**) Fear ratings to CS+/CS− measured using a Visual Analogue Scale during expression in a second experiment.

To break down this significant interaction, planned comparisons demonstrated that in the placebo group, there were significantly higher SCRs for CS+ than CS−, *t* (23) = 3.612, *p* = 0.001, *d* = 0.73, however this was not the case in the tyrosine group, *t* (23) = 0.427, *p* = 0.674, *d* = 0.13. When comparing SCRs for CS+ between the placebo and tyrosine group, significantly higher fear responses occurred for the placebo than tyrosine group, *t* (44) = 2.914, *p* = 0.006, *d* = 0.92, whilst no significant differences were reported for CS−, *t* (44) = 0.470, *p* = 0.641, *d* = 0.13 (see Fig. 1B). These findings therefore demonstrate that tyrosine impairs fear expression.

### Control measures: SCR magnitudes during UCS

We checked whether tyrosine effects on fear conditioning could be ascribed to an association between CS+ and UCS, as opposed to unspecific systemic effects on UCS alone. We therefore conducted a drugs × time mixed ANOVA of the responses to the UCS.

There was a significant main effect of time (F (1, 44) = 16.89, *p* < 0.001, η^2^ = 0.277). This indicates that SCRs to UCS decreased over time regardless of the drug (see Fig. 2A). Importantly, there was neither a main effect of drugs (F (1, 44) = 0.001, *p* = 0.985, η^2^ = 0.000) nor a time × drugs significant interaction (F (1, 44) = 0.632, *p* = 0.431, η^2^ = 0.014).

**Figure 3:**
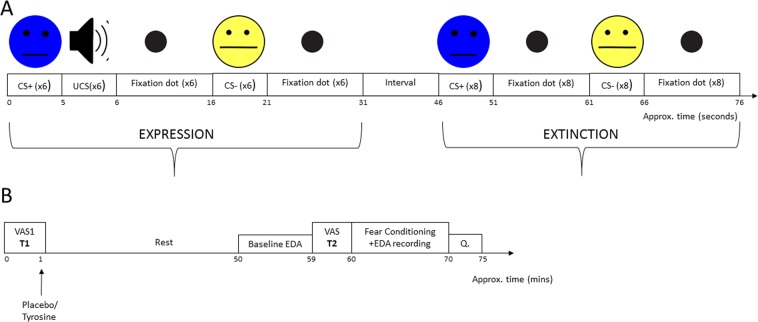
(**A**) Schematic representation of fear conditioning task. Stimuli were presented in a pseudorandomized order. In brackets (x6 or x8) indicate the number of CS+ or CS− pairings during expression and extinction. Time is shown in seconds. (**B**) Summary of experimental procedure. Q = questionnaire to assess the double-blind efficacy of placebo/tyrosine administration. VAS = visual analogue scale. T1 = time 1. T2 = time 2. Time is shown in minutes.

### Control measures: mood, double blinding efficacy and fear ratings

Transient changes in mood state have been demonstrated to influence fear learning expression^34^. We therefore administered a visual analogue scale (VAS) questionnaire before and after drug administration (but before fear conditioning: see Fig. 3B). We also checked whether the double blinding procedure of tyrosine/placebo administration had been effective.

There was neither a significant main effect of drugs on mood scores (*F* (1, 44) = 0.004, *p* = 0.951) nor a drugs × time interaction on mood scores (*F* (1, 44) = 1.54, *p* = 0.220). The probability of participants guessing the correct drug (placebo or tyrosine) was below chance at 39% (42% for placebo and 36% for tyrosine).

In a separate control experiment, fear/valence ratings to CSs (CS+ and CS−) were evaluated using an additional computerized VAS with values ranging from 0 (no fear/stress/tension) to 100 (maximal fear/stress/tension) (see Procedures), as per administered by Haaker *et al*.^35^.

There was neither a significant main effect of block on VAS fear ratings (*F* (1, 17) = 3.73, *p* = 0.070), nor a block × stimuli significant interaction (*F* (1, 17) = 1.332, *p* = 0.264). Importantly, there was a significant main effect of stimuli (*F* (1, 17) = 417.4, *p* =  < 0.001), demonstrating robust conditioning effects to the CS+ (Fig. 2B).

In a second control experiment, participants (N = 21) were asked to rate the perceived aversiveness of the UCS based on three dimensions, *unpleasantness, intensity*, and how *startled they were* (see Procedures), as per previously published protocols^36^. Participants rated the UCS as *unpleasant* (M = 76.2, SD = 6.1), *intense* (M = 68.4, SD = 6.6), and were *moderately* to *strongly startled* (M = 74.2, SD = 8.2), demonstrating the overall perceived aversiveness of the UCS (Fig. S1).

### SCR magnitudes in extinction

As tyrosine blocked fear expression, we cannot interpret its effects in extinction and we report these results only for completeness in the supplementary file, together with its Figure. (S2).

## Discussion

Numerous studies over the last decade have demonstrated an involvement of dopamine and norepinephrine in both fear acquisition, expression and extinction. Most of these investigations have been carried out in rodents. In humans, work has been limited to manipulations of catecholaminergic activity post fear acquisition learning and hence focusing on extinction, extinction consolidation and reconsolidation processes only.

This work provides novel evidence that a catecholaminergic precursor administered before conditioning abolishes fear expression. Specifically, augmentation of putative dopamine and norepinephrine levels by tyrosine administration rendered SCR responses to CSs in expression indistinguishable from one another (i.e. SCRs magnitudes to CS+ and CS− were approximately equal and cancelled each other out: see Fig. 1A,B). Moreover, SCRs to CS+ were greater in the placebo group than in those administered tyrosine. Importantly, tyrosine administration did not alter processing of UCS information (see Fig. 2A) demonstrating that tyrosine selectively weakened CS+ UCS associations. Moreover, there were no unspecific systemic changes of tyrosine administration on measures of mood and alertness, which may have affected fear expression.

It is worthwhile discussing the learning curve observed in the fear instructed paradigm. As can be seen in Fig. 1, fear responses to CSs decreased over time. Although this pattern is common in human studies^20,37–39^, it is the opposite in animals’ studies^4,8^, in which fear responses increase over time.

There are a number of potential factors that can account for this phenomenon. These factors include the type of CS used, UCS identity and intensity, the length of inter-trial and inter-stimulus interval, the reinforcement rate, the trial number and order, the CS/UCS duration, the type of instructions (e.g. explicit CS-UCS contingency or not), whether the UCS was experienced prior to testing (i.e. UCS calibration), and variations in acquisition procedures (e.g. single-cue vs differential protocols; multiple-cue protocols) (for a more thorough review, see^23^).

Our findings that tyrosine abolished fear expression are important and surprising given that in the animal literature, increased fear acquisition and expression has been reported when a dopaminergic D1 agonist was administered^8,9^ or abolished when D1/D2 receptors were blocked^3–6^. Similar results have been reported with respect to manipulations of norepinephrine^14–17^. These contrasting findings may partially be explained by methodological differences in manipulating dopaminergic and norepinephrine activity postsynaptically in animal studies and presynaptically in the current study. It is also plausible, though speculative at present that tyrosine administration may have interfered with the process of prediction error signalling. The strength of this signal would have been greatest in early trials, when the occurrence of the UCS is most surprising^40^ and learning (of the CS-UCS pairing) is most likely to occur^41,42^. Midbrain dopaminergic neurons have long been known to convey a prediction error signal for rewards^43,44^, but more recent evidence also demonstrate a role for aversive events^45–47^. Nevertheless, because we employed an instructed fear conditioning protocol, the UCS presentation in the early trials should not have been very surprising, given that participants had been made aware of what to expect. Therefore, it is perhaps more plausible to suggest that differences between the animal literature and our current finding, relate to the use of an instructed fear paradigm. Previous research, in humans, has suggested that uninstructed fear conditioning paradigms (as in most animal studies) recruit a differential neuronal circuitry to that of instructed fear studies^48^.

With respect to human studies, our findings cannot directly be compared to previous investigations, especially those that manipulated dopaminergic activity *after* fear acquisition had taken place and investigated extinction and extinction consolidation. Onur *et al*. manipulated norepinephrine levels *before* conditioning. In their study, administration of the NE reuptake inhibitor reboxetine induced an amygdala response bias towards fear signals^49^. Similarly, Visser *et al*. reported a rise in salivary-amylase levels (a marker of markers of noradrenergic activation) prior to fear conditioning which correlated with fear consolidation expression^50^. Taken together, in humans, increasing catecholamines (using tyrosine) before fear conditioning impairs fear expression whilst pharmacological augmentation of norepinephrine before conditioning enhance fear acquisition. In animals, targeted manipulations of both dopamine and norepinephrine that either increase or decrease their release before fear conditioning enhance and impair acquisition learning respectively.

The choice of administering tyrosine instead of L-Dopa as in past studies was motivated by a number of reasons. Firstly, conversion of tyrosine into dopamine (and other catecholamines) is restricted by the transporter shared with other amino acids and by the rate-limiting TH enzyme. L-Dopa, on the contrary, is not affected by these and its administration would result in greater concentrations being converted to catecholamines^31^. However, given the well-established inverted U relationship between dopamine concentration and performance^51^, it is foreseeable that L-Dopa would be more likely than tyrosine to shift participants at the right end of the curve. This would be particularly the case in individuals who are homozygous for the Met/Met allele on the Val(108/158)Met COMT polymorphism, and possess higher baseline dopaminergic activity^52^. Secondly, an additional advantage of administering tyrosine is that it produces fewer and less severe side effects than L-Dopa (e.g. nausea, insomnia, psychosis)^53^.

As tyrosine is the precursor for dopamine, norepinephrine (noradrenaline) and epinephrine it can be concluded that these neurotransmitters are involved in fear expression. Finally, it is worth considering the implications of our data in the context of the pathogenesis of anxiety disorders. Two large meta-analyses^54,55^, looked at fear conditioned responses of healthy and anxiety patients to CS+, CS− and the conditioned response (CR) difference between (CS+) - (CS−). Contrary to theories by Davies and colleagues^56^, who suggested that healthy individuals would produce higher CRs to (CS+) than (CS−) compared to anxious individuals in acquisition, the data from the meta-analyses demonstrated that this was not the case. Similarly, the meta-analyses found no significant differences in responding to CS+ between healthy and anxious patients. Importantly, it was found that responding to the conditioned safety cue (CS−) was higher in anxious patients compared to healthy controls. These results suggest that any pharmacological agent with anxiolytic properties should reduce conditioned responses to CS− in anxious patients to match those of controls, and that in healthy participants the pharmacological agent would reduce even further conditioned responses to the safety cue compared to a placebo group.

Based on our data, we conclude that tyrosine did not have such anxiolytic properties, but rather, that impaired fear expression responses.

### Limitations and future directions

Although we established that catecholamines contributed to fear expression, our study did not set out the reveal the neuronal pathways that would have determined this effect. Therefore, future investigations using imaging techniques (e.g. PET) would be required to understand how changes in dopamine/norepinephrine in brain regions of interest regulate fear expression (e.g. amygdala-prefrontal cortex). Furthermore, to obtain greater specificity with respect to dopamine for example, one would need to co-administer a noradrenergic blocker such as Clonidine together with tyrosine/L-Dopa, or target D1/D2 receptors. More crudely, reducing catecholaminergic neurotransmission using the acute phenylalanine/tyrosine procedure^57^ before conditioning, would provide a potential counter test for the effects of manipulating catecholamines on fear expression.

In conclusion, our results provide evidence that manipulating catecholaminergic tone before conditioning in humans can suppress fear expression.

## Method

### Participants

Participants consisted of 46 university students (M = 20.4, SD = 1.7; 25 females and 21 males). The study was approved by the ethics committee of Sheffield Hallam University (SHU) and complied with the Declaration of Helsinki. All study methods were performed in accordance with SHU guidelines. Informed consent was obtained for all participants before testing could take place. Exclusion criteria included: those suffering from cardiac, hepatic, renal and neurological disorders and individuals with a history of alcohol or drug addiction, or psychiatric illness. Individuals having a history of taking tyrosine supplements were also excluded.

### Drug administration

Participants received either 2.0 g of l-Tyrosine (supplied by BulkPowders Ltd.) or 2.0 g of the placebo microcrystalline cellulose (Redwells Creative Limited,UK) dissolved in 400 ml of orange juice as per previously published protocols^58,59^. Dosages greater than 2.0 grams have been shown not to provide additional benefits given that the rate-limiting tyrosine hydroxylase (TH) enzyme is already close to saturation under normal circumstances^60^. Peak plasma tyrosine levels have been reported to occur between 60 and 120 minutes post-ingestion^61^. In rats, increases in prefrontal dopamine concentrations can be observed 60 minutes following tyrosine administration^62^.

### Electrodermal activity recording

Physiological recording were obtained using a BIOPAC MP150 data acquisition unit (Biopac Systems Inc., Santa Barbara, CA), and Acq*Knowledge* 4.4 software. Electrodermal activity (EDA) data were collected at 2000 samples/sec. Two disposable latex-free electrodes (EL507, BIOPAC) containing isotonic gel were attached to the distal phalanges of the index and middle fingers of the non-dominant hand. The EDA signal was then enhanced by a wireless BioNomadix ® amplifier. Skin conductance responses (SCR) threshold was set at 0.01 microSiemens (µS) with SCR onsets and peaks (i.e. a measure of change in tonic EDA) counted as being event related if occurring between 1–4.5 seconds following stimulus (CS+/CS−/UCS) onset. Data transformation of SCR magnitudes was applied using the square root (√SCR) as in previously published protocols^63^. Participants who did not produce a measurable conditioned response (i.e. classified as CS+ = SCR amplitude not reaching the 0.01 threshold across the whole conditioning session) (8/56), a similar criterion to previous reports^64^, were discarded from the analyses as those with poor electrode contact/motion artefacts (2/56), giving us a remaining sample of 46 volunteers. Of those 46, 24 participants were in the placebo condition and 22 in the tyrosine. To confirm that the overall results were not dependent on the exclusion of these participants, we rerun these analyses excluding only those due to poor recording (2/56) and those who did not respond to the UCS (2/56) (i.e. not the outcome measure), giving us a remaining sample of 52 volunteers, 26 placebo and 26 tyrosine. These data (see Supplementary info) confirm the conclusions reached in the results section.

### Fear instructed task

The conditioning task was programmed in OpenSesame^65^. Visual stimuli consisted of blue and yellow circles either predicting an aversive stimulus (CS+) or a neutral fixation dot (CS−). These were presented in a counterbalanced (i.e. half of the participants had the blue circle as CS+ and the other half had the yellow circle as the CS+) and randomized order (i.e. the CS+/CS− sequence varied for each participant). Before testing began, on screen instructions told participants which of the two CSs would be paired with the aversive stimulus (UCS) (as in “In this experiment, the following stimulus [image 1 shown] will be followed by a loud beep, whilst another stimulus [image 2 shown] will be followed by a neutral fixation dot [image 3 shown]. You are not required to perform any actions but to remain as still as possible throughout the duration of the experiment. The experiment will last approximately 8 minutes”) which consisted of a loud beep (75 db measured using an audiometer) lasting 1000 ms delivered through headphones. Moreover, participants were not exposed to the UCS prior to testing. In a previous pilot study, the UCS reliably elicited SCRs in over 90% of the trials.

The task was divided into an expression and an extinction phase. Each CS presentation lasted 5000 ms with an intertrial interval (ITI) of 10000 ms consisting of a fixation dot. During expression, there were 6 pairings of CS+ and UCS (as in CS+ for 5000 ms, followed by UCS for 1000 ms and fixation dot [ITI] for 10000 ms) and 6 of CS− (as in CS− for 5000 ms, followed by the same fixation dot for 10000 ms). The duration of the ITI was chosen based on previous reports^19^ and permitted recovery of the SCR. In the extinction phase, both stimuli were unpaired (CS+ and CS− followed by the fixation dot) and presented 8 times each. The task lasted approximately 8 minutes. A schematic illustration of the task is shown in Fig. 3A.

### Control measures: mood, double-blinding efficacy and fear ratings

We checked for the potential mood effects induced by tyrosine intake by administering a computerized adaptation of the visual analog scale (VAS) which was programmed and run in PEBL^66^, and has previously been used by our research group^67–69^. The scale consists of seven dimensions (e.g. boredom, sadness, relaxation, happiness, stress, alertness, calmness) of mood/alertness. The double-blinding efficacy of placebo/tyrosine administration was checked by a questionnaire (i.e. “Please circle whether you think you received a tyrosine containing drink (experimental) or a placebo, non-tyrosine containing drink (control)”) given to participants at the end of the experiment.

Fear ratings for CS+/CS− were measured in a separate cohort of participants (n = 18) using a computerized VAS as per previous research groups^35^. Briefly, after the 3^rd^ CS+/CS− pairing, and at the end of the last trial (trials 6 for both CS+ and CS−), participants rated each CS on a scale ranging from 0 (no fear/stress/tension) to 100 (maximal fear/stress/tension).

Additionally, we measured in a third cohort of participants (n = 21) their perceived aversiveness to UCS presentation using a similar approach to that of Hermans *et al*.^36^. Note that, as reviewed elsewhere^2^, there is no agreed consensus on the precise procedural approach to measuring aversiveness (i.e. the type of visual/verbal scale used) and which criterion constitutes the threshold for the UCS being perceived as aversive, with minimum (aversiveness) scores ranging from 5 out of 10 to 8 out of 10. Using a computerized VAS, participants rated the UCS for three characteristics; the first was the *unpleasantness* of the stimulus, with a score of 0 being described as *pleasant*, 50 *neutral*, and 100 *unpleasant*. The second was the *intensity* of the UCS, with 0 being classified as *light*, 50 *intense* and 100 *intolerable*. The third was how *startled* participants responded to the UCS, with 0 being labelled *not at all*, 50 *moderately*, and 100 *very strongly*. Participants were presented with the UCS six times, using similar timing intervals between each UCS as in the fear instructed study, but without CS+/CS−. After the last UCS, participants completed the VAS.

### Procedure

This was a double-blind, placebo-controlled, mixed design experiment. Participants were required to attend a session lasting approximately 75 minutes. After screening for eligibility, participants were instructed to refrain from eating/drinking for a minimum of 3 hours. This is to reduce competition from other amino acids that share the same transporter^70^. They first signed a consent form followed by the mood questionnaire (VAS; time 1), and were then randomly assigned to receive either tyrosine or placebo. Participants were then asked to rest for 50 minutes following tyrosine/placebo intake, at the end of which, EDA electrodes were attached to the participant’s index and middle finger. At 59 minutes (post tyrosine/placebo consumption), the VAS was completed for a second time (time 2). At 60 minutes, fear conditioning and EDA recording began. Participants were instructed to remain as still as possible during testing to reduce motion artefacts. Following this, participants completed the tyrosine/placebo double-blind questionnaire, and were debriefed (see Fig. 3B).

### Statistical analyses

Sample size was estimated using the following parameters: power 0.8, alpha 0.05, mixed design ANOVA containing 2 groups, 2 repeated measurements and a large f^2^ effect size of 0.4 (G*Power 3.1.9.2, Germany). The size of the effect size was based on the results of previous reports which looked at fear conditioning responses (SCR) using another catecholaminergic precursor (l-DOPA)^20^.

We performed a number of analyses on SCR measurements. First, we calculated expression responses on trial by trial basis (i.e. across the 6 CS+ and CS− pairings). The following variables were considered: Drugs (placebo and tyrosine), Trials (trial 1 to trial 6) and CS identity/stimuli (CS+, CS−). SCRs were measured 1–4.5 seconds following stimulus onset (CS+/CS−) which terminated after 5 second and hence did not include the UCS (which was presented between 5–6 seconds following CS+ onset), as in previous reports^22,64^, to isolate acquisition effects specific to the CS + and not confounded by the UCS.

Second, we run a Drug (placebo and tyrosine) × Time (early trials, and late trials) ANOVA of the responses to the UCS, here also measuring SCRs to UCS in the 1–4.5 seconds following stimulus (UCS) onset. This type of analysis has been done by other research groups^71^, and allows to capture UCS encoding in acquisition.

## Supplementary information

Supplementary file
