# Supplementary material for: Fear expression is suppressed by tyrosine administration
Source: Sci Rep. 2019 Nov 5;9:16073. doi: 10.1038/s41598-019-52610-x (PMC6831598; doi:10.1038/s41598-019-52610-x)
Supplement: Supplementary file 1 — Supplementary file [file 41598_2019_52610_MOESM1_ESM.docx]

Fear expression is suppressed by tyrosine administration

Alessandro Soranzo, Luca Aquili^,^*

Department of Psychology, Sociology and Politics, Sheffield Hallam University, Sheffield, UK

* Corresponding author

**Supplementary information**

**Results**


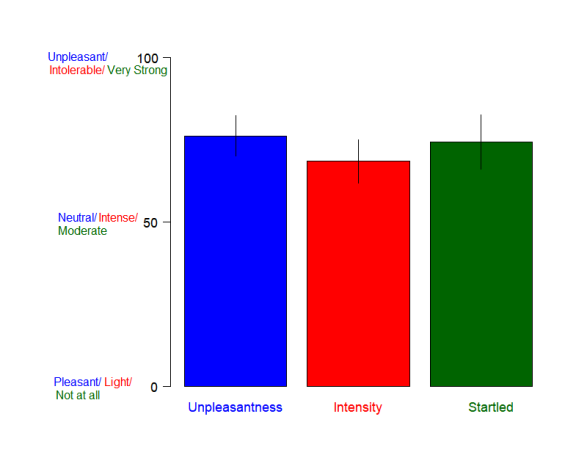


Fig S1. Aversiveness ratings to UCS measured using a Visual Analogue Scale (0-100) in a third experiment. Three characteristics of aversiveness were measured: unpleasantness (blue bar), intensity (red bar), and how startled participants were (green bar). Vertical lines represent standard deviation (+/- 1).

*SCR magnitudes in expression (no UCS responses criterion)*

A 2x2x6 factorial mixed analysis of variance was conducted on SCR magnitudes during the acquisition phase using an exclusion criterion that only removed those with motion artefact/poor physiological recording (2/56) and those with an absence of responses to UCS (2/56).

The between group variable, *drugs*, having two levels (placebo, tyrosine) the first within subject variable, *stimulus*, having two levels (CS+, CS-), and the second within subject variable, *trial,* having six levels (trial 1-6). There was neither a significant main effect of drugs, *F* (1, 50) =1.265, *p*=0.266, _Ƞ_^2^=.025, nor a main effect of stimuli, *F* (1, 50) =1.912, *p*=0.173, _Ƞ_^2^=.037, nor a trial x drugs, *F* (5, 50) =2.025, *p*=0.076, _Ƞ_^2^=.039, nor a trial x drugs x stimuli significant interaction, *F* (5, 50) =.724, *p*=0.606, _Ƞ_^2^=.014.

There was, however, a significant main effect of trial, *F* (1, 50) =9.447, *p*<.001, _Ƞ_^2^=.159, a significant trial x stimulus interaction, *F* (3.8, 191) =3.231, *p*=0.015, _Ƞ_^2^=.061, and most importantly, a significant drugs x stimuli significant interaction, *F* (5, 50) =4.524, *p*=0.038, _Ƞ_^2^=.083).

To break down this significant interaction, planned comparisons demonstrated that in the placebo group, there were significantly higher SCRs for CS+ than CS- , *t* (25) = 3.542, *p*=0.002, however this was not the case in the tyrosine group, *t* (25)= .428, *p*=0.672. When comparing SCRs for CS+ between the placebo and tyrosine group, significantly higher fear responses occurred for the placebo than tyrosine group, *t* (50) = 2.997, *p*=0.004, whilst no significant differences were reported for CS-, *t* (50) = .258, *p*=0.798. These findings confirm the data reported in the results section of this manuscript using a different exclusion criterion (Fig 2A and 2B) and therefore demonstrate, once again, that tyrosine impairs fear acquisition learning.

*SCR magnitudes ([CS+] - [CS-] in extinction)*

Following acquisition, the fear conditioning task had an extinction phase, during which CS+ was presented eight times but was never paired with the UCS. The CS- was also presented eight times. Responses were analysed on the basis of whether these occurred during the early stages of extinction (Early Trials: trial 1 and 2= averaged) and later stages (Late Trials: trial 7 and 8= averaged).

A 2x2 mixed analysis of variance was conducted on SCR magnitudes during the extinction phase. There was neither a main effect of drugs (F (1, 44) = 1.942, *p*=.170, _Ƞ_^2^= .042) nor a main effect of time (F (1, 44) =0.023, *p*=.880, _Ƞ_^2^=.001), nor a time x drugs significant interaction (F (1, 44) = 2.555 *p*=.117, _Ƞ_^2^= .055) (see Fig. S1).

**
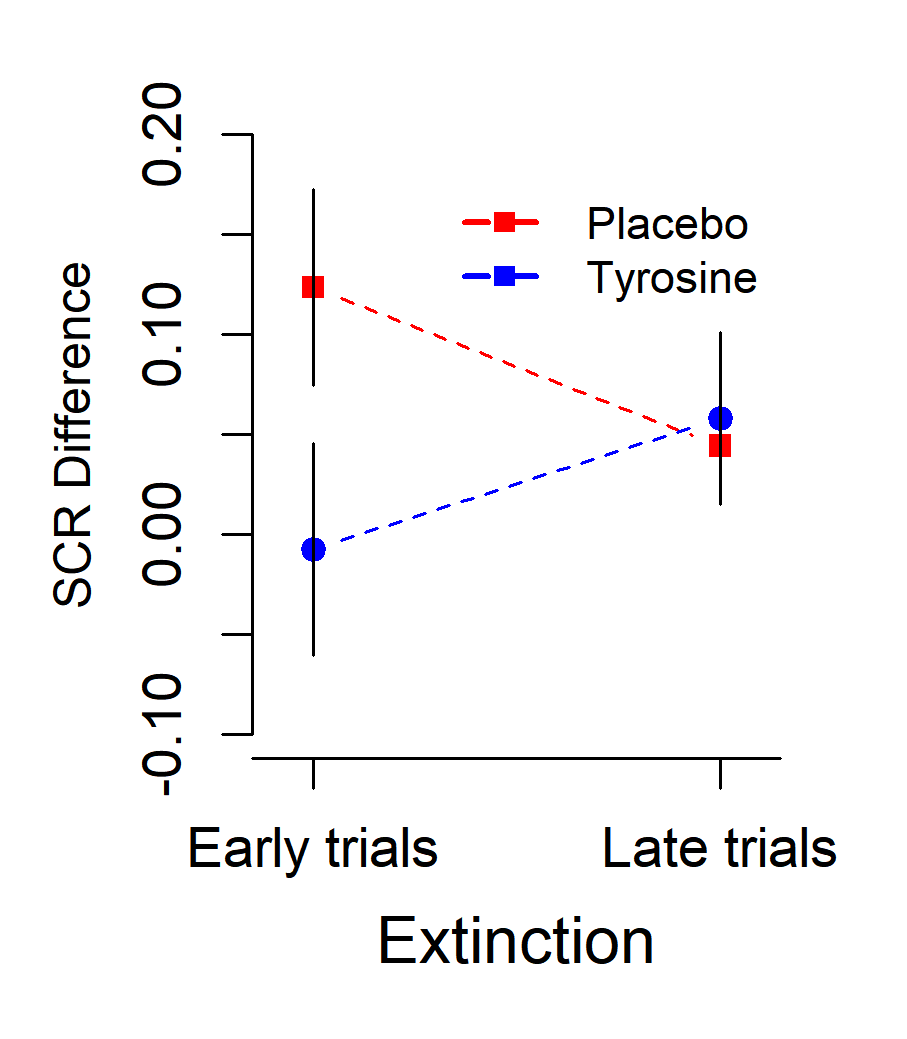
**

Fig.S2. Line charts representing skin conductance responses measured in magnitudes during early, and late trials, based on the SCR difference between CS identity ([CS+] -[CS-]), in the placebo and tyrosine groups during the extinction phase.. Vertical lines represent standard error of the mean.

*Frequency of CS+/CS- presentation as first stimulus at trial 1*

Although we found neither a trial (1-6) x drugs (placebo, tyrosine) x stimuli (CS+/CS-) significant interaction nor a significant trial x drug interaction (using CS+ as only measure), we wanted to exclude the possibility that the visually apparent greater SCR responses to CS+ at trial 1 in placebo participants compared to the tyrosine group (see Fig. 1A) was not caused by the CS+ being presented more frequently as the first stimulus in the sequence for the placebo group than for the tyrosine group. If this were the case, it would suggest that the greater SCR to CS+ in the placebo participants could be attributable to an attentional orienting SCR.

To answer this question, we run a Chi-Square test. There was no significant association between the condition in which a participant was in (i.e. placebo or tyrosine) and the frequency of CS+/CS- occurring as the first stimulus at trial 1, [_x_^2^ (1) =.300, *p*=0.584]. The number of participants in the placebo condition which were exposed to CS+ as the first stimulus at trial 1 was 9/24, and 15/24 for CS-. For the tyrosine participants, 10/22 experienced CS+, and 12/22 CS-.

Overall, these data confirm that the relative frequency of CS+/CS- presentation as the first stimulus at trial 1, was not a determinant of higher SCR to the CS+ in the placebo group, and this excludes a purely attentional component as an alternative explanation to the observation.

*Effect of time on SCR responses*

To test the effect of time on a differential SCR response in placebo/tyrosine participants, we reanalysed the data by only looking at responses to CS+. Therefore, we run a trial (1-6) by drug (placebo, tyrosine) mixed factorial ANOVA.

In agreement with our initial analysis, there was no significant trial x drug interaction [*F* (3.8, 167.6) =1.751, *p*=0.144, _Ƞ_^2^=.038], and a main significant effect of drug [*F* (1, 44) =8.494, *p*=.006, _Ƞ_^2^=.162]. These data confirm that tyrosine suppressed fear expression responses (to CS+) throughout conditioning, and not on a specific trial.
